# Repeatability and Reproducibility of a Saccadic Eye Movement Time Test

**DOI:** 10.3390/jcm14207170

**Published:** 2025-10-11

**Authors:** Antonio Ríder-Vázquez, Estanislao Gutiérrez-Sánchez, Daniel Velasco-Olea, Clara Martinez-Perez, María Carmen Sánchez-González

**Affiliations:** 1 Department of Physics of Condensed Matter (Optics Area), University of Seville, Reina Mercedes S/N, 41012 Seville, Spain; antonioriderv@gmail.com (A.R.-V.); msanchez77@us.es (M.C.S.-G.); 2 Department of Surgery (Ophthalmology Area), University of Seville, Doctor Fedriani S/N, 41009 Seville, Spain; egutierrez1@us.es; 3 Centro de Optometría Internacional (COI), Chile Nº10, 28016 Madrid, Spain; d.velasco@coi-sl.es; 4 Applied Physics Department (Optometry Area), Facultade de Óptica e Optometría, Universidade de Santiago de Compostela, 15705 Santiago de Compostela, Spain

**Keywords:** saccadic eye movements, sports vision, intra- and interexaminer repeatability, visual performance, optometry

## Abstract

**Background/Objectives**: Reliable and objective assessment of saccadic duration is crucial in sports vision, yet standardized clinical tools remain scarce; therefore, this study evaluated the intraobserver and interobserver repeatability of saccadic time measurements using COI-SV^®^ software, and analyzed the influence of age and sex. **Methods**: Saccadic duration was assessed in 78 participants using a 20/40 Snellen letter stimulus appearing in four directions (up, down, left, right) at two distances. The shortest response time per direction was recorded. General mean values (total, vertical, horizontal, short, long, and ratios) were calculated. Repeatability was evaluated through a protocol of four test repetitions (two intrasession and two intersession with different examiners). ANCOVA and Pearson correlation assessed sex and age effects. Repeatability indices and Bland–Altman plots were used to determine agreement. **Results**: Regarding sex, there were no significant differences between men and women. Saccadic duration showed a direct relationship with age (*p* < 0.05), indicating that older participants had worse saccadic time values (longer times). Overall, intraexaminer repeatability was poor, whereas interexaminer reproducibility was between fair and good. Bland–Altman analysis showed limits of agreement ranging from −159.0 to 220.3 milliseconds (ms) for specific time values and from −87.0 to 122.52 ms for general values, which may be useful in clinical practice. **Conclusions**: The study shows that the COI-SV^®^ software provides moderate to good interexaminer reliability and poor to acceptable intraexaminer repeatability of saccadic duration measurements, indicating that further refinement and validation are needed before considering clinical implementation.

## 1. Introduction

Sports vision encompasses a set of assessment and training approaches designed to maintain and improve visual abilities, with the goal of enhancing performance in athletic activities [1]. Despite its importance, visual function and sensory information derived from vision are often overlooked and remain unfamiliar to many athletes, coaches, and even healthcare professionals. Certain visual skills, such as static and dynamic visual acuity, contrast sensitivity, depth perception, peripheral awareness, and visual–motor reaction time, are essential across nearly all sports [2]. Additionally, recent research has shown that elite athletes tend to have superior visual abilities, such as a wider visual field and better eye movement control [3].

Several studies using speed-based visual tests, such as the King–Devick (K–D) test, have demonstrated that athletes tend to outperform non-athletes in rapid eye movement and visual–motor tasks. For example, in collegiate athletes, Galetta et al. showed that K–D scores significantly worsened after concussion and reported strong test–retest reliability under normal play conditions, supporting its usefulness in athletic populations [4]. In adolescents and youth players, the K–D test has also been applied to assess baseline differences between competitive and recreational athletes, consistently showing faster times in higher-performing groups [5].

Regarding the Developmental Eye Movement (DEM) test, although originally designed for children, several studies have evaluated its reliability for vertical and horizontal times and reported high coefficients for these components, while ratio scores and error counts showed only moderate to low reliability [6,7]. In addition, Facchin et al. recently demonstrated in Italian adults that age significantly influences DEM times (vertical, adjusted horizontal, and ratio), though not error rates [8]. Beyond these cohorts, studies in basketball, baseball, and cricket players have also highlighted the role of saccadic speed and accuracy in ball tracking, anticipation, and decision-making, confirming that elite athletes often exhibit superior performance in these tasks [9,10,11,12]. Recent evidence using the Saccadic Eye Movement Speed Test further supports these findings, showing that faster saccadic execution is associated with improved athletic performance [13].

Although these tests have not yet been widely used in structured sports research on an international scale, their application is more common in developed countries, whereas in developing regions vision training programs and objective evaluations remain limited.

To achieve optimal performance, it is crucial that an athlete’s visual system effectively gathers sensory input. This input is then processed by the brain, which filters the most pertinent information to enable an appropriate motor response. However, standardized measures are lacking, so visual processing capabilities are often overlooked in clinical evaluations [14].

Although sports optometry has been studied for decades, it has gained increased scientific attention and development only in recent years, highlighting the need for more high-quality research. There is a notable gap in the literature regarding three key areas that are fundamental to sports performance: perceptual speed, ocular motility, and eye-hand coordination [15].

Regarding ocular motility, standardized measures for saccadic evaluation are lacking. Saccades involve rapid eye movements that humans make to direct the fovea to an object of interest, typically requiring both action and perception. These processes are often studied separately and could offer valuable insights into how the brain pathways for action and perception interact [16].

In sports vision, saccadic duration evaluates how quickly an athlete can perform a saccade and interpret an object of interest in the shortest possible time. This allows comparisons between athletes and provides critical insights, since even minor perceptual differences can impact performance. Most motility tests involve subjective observation of eye movements, using rating scales such as those from The Southern California College of Optometry or the Northeastern State University College of optometry. However, due to their subjective nature, these scales can be challenging for inexperienced clinicians to apply effectively and are largely unsuitable for the sports vision field.

Objective tests are scarce. Some, like the Visagraph III or Readalyzer, are costly and not widely used. The most commonly used tests follow a visual–verbal format, including the Pierce Saccadic Test, King-Devick, New York State Optometric Association King-Devick Test, and the Developmental Eye Movement (DEM) test. These tests share a similar design: patients are asked to read aloud a series of numbers as quickly as possible, with their response times and number of errors compared to age-normed values. Among these, the DEM is the most widely used and is unique in differentiating between saccadic dysfunction and number-naming difficulties. However, these tests are designed for reading tasks, which differ significantly from the demands of sports. Moreover, they are generally used with children, making them less valid for sports vision applications [17,18,19,20].

On the other hand, eye-tracking technology has been widely used in research to obtain more precise measurements of saccadic movements, but standardized protocols are lacking. Most research focuses on the correlation between saccadic performance and reading abilities [21], particularly in children with intellectual disabilities [22] or on the relationship between saccadic function and mental disorders [16,23], especially in degenerative pathologies [24,25] and patients with a history of concussion [26,27]. These studies generally conclude that saccadic slowing is common in neurological disorders and reading difficulties.

Additionally, some research indicates that the longer a person spends on a task, the slower their saccadic speed, highlighting the critical role of fatigue [28], which may have implications for sports vision. However, most eye-tracking studies have primarily focused on motor outcomes such as latency, velocity, or accuracy of saccades, without integrating perceptual or cognitive processing demands [22,23]. This limitation is relevant because sports performance requires not only precise ocular motor control but also rapid visual discrimination and decision-making. The only study addressing the interaction between perception and ocular motility is the one made by Stan et al., [16] where participants completed two tasks: a simple saccadic task (SST) and a dual saccade-decision task (DST). Results indicated poorer performance in the DST, suggesting the added complexity affects saccadic efficiency.

Age and sex are important factors in evaluating saccadic performance, but their influence varies depending on the specific saccadic parameter assessed. For example, research shows that saccadic latency and peak velocity tend to decline in older adults (aged 60 to 90) compared to younger adults and children, suggesting an age-related slowing of oculomotor response times [27,29]. However, other studies using behavioral tests like the DEM (which emphasize visual–verbal integration and indirectly reflect saccadic efficiency) did not find significant differences in duration or accuracy between younger adults and children [30]. Interestingly, normative data from the DEM indicate that older children (around 13 years) tend to outperform younger children (around 6 years) [31], likely reflecting maturational improvements in oculomotor control and cognitive processing. Regarding sex, there is a notable lack of research and further studies are needed to determine possible differences between males and females in saccadic performance.

Although saccadic eye movements have been studied previously, this specific variable has not been extensively explored using digital, time-sensitive tools in the context of sports vision, making it a novel focus within the field. Consistent measurement methods, where different examiners achieve similar results using the same device, are crucial for reliable assessments [32]. Therefore, this study aimed to evaluate the intra- and inter-observer repeatability of a method designed to assess saccadic duration using COI-SV^®^ software, as well as to analyze the potential influence of sex and age on the results.

## 2. Materials and Methods

### 2.1. Study Design

A prospective study was conducted at the facilities of “Centro de Optometría Internacional (COI)” in Madrid, Spain, from May to June 2023. A total of 79 participants (42 men and 37 women) were recruited for the study; however, one man was excluded for not meeting the inclusion criteria, yielding a final sample of 78 participants (41 men and 37 women). All participants provided informed consent, in accordance with ethical standards. The study was carried out in compliance with Declaration of Helsinki (1964) and was reviewed by the Research Ethics Committee of the University of Seville.

### 2.2. Participant Enrolment

Sample size calculations were performed using the Granmo calculator, version 7.12 (Institut Municipal d’Investigació Médica, Barcelona, Spain) [33]. Based on the parameters set for the study, it was determined that a sample of 49 participants would be sufficient to achieve 80% power, with an alpha risk of 0.05 and a beta risk of 0.2 in a two-sided test.

Eligible participants for the study were aged between 18 and 65 years and had a distance binocular and monocular visual acuity (VA) of 20/25 or better with their distance correction. Participants were excluded if they met any of the following conditions: presence of any pathological condition that could lead to visual field restrictions, affectation of reaction times and/or affectation of ocular motility, use of chronic medication that could potentially influence reaction times, use of acute medication that could affect reaction times in the 24 h prior to the test and consumption of alcohol, drugs or any other substances that might influence test performance within the 24 h prior to testing.

Participants were recruited through the dissemination of advertisements. These criteria were established to ensure that the study population represented healthy adults without ocular or systemic conditions likely to interfere with oculomotor performance.

### 2.3. Saccadic Duration Test

This test provides an objective method to measure saccadic eye movements by assessing the time required to perform saccades in response to peripheral stimuli. Visual stimuli consisted of 20/40 Snellen letters presented at 1 m distance. A central fixation letter was followed by peripheral letters at 50 ms intervals, beginning 400 ms after fixation onset, and letters were randomly displayed in four directions (up, down, left, right) and at two eccentricities: short (15° horizontal; 10° vertical) and long (30° horizontal; 20° vertical). The design prevented reading with extrafoveal vision [34] and the shortest response time in each direction was recorded.

Once the 8 specific values were recorded, general mean values were calculated, including total, vertical, horizontal, short, long and ratios (horizontal–vertical and short–long).

Saccadic duration was measured using the COI-SV^®^ digital software. Developed by “Centro de Optometría Internacional” (Madrid, Spain), this software includes diagnostic and treatment tests specifically tailored for sports vision and enables a digital and time-precise measurement of saccadic performance [35]. It has been previously applied in similar studies [2,36], but its reliability for temporal measurements required direct evaluation, which was one of the main aims of the present study. All participants completed a pre-test with 500 ms presentations to ensure understanding and minimize learning effects, which was not included in the data analysis. All instruments were calibrated before data collection.

To minimize bias, examiners gave identical instructions to participants: stand on the line (1 m away from the screen) and stay alert, as letters are going to appear. Initially, focus on the letter in the center, and when the other letter appears, quickly look at it and say which letter you saw. The letters may appear above, below, to the right, or to the left of the central letter. The test then commenced.

### 2.4. Test and Re-Test Methodology

To assess the repeatability of the measurement method, the protocol was designed so that each participant repeated the tests twice on two different days, totaling four trials under identical conditions [19,35,37,38,39]. This design enabled the evaluation of intra-session and inter-session repeatability, as well as interexaminer reproducibility, following approaches previously applied in reliability studies in vision science [19,35,37,38,39]. On the first day, participants completed preliminary tests and a brief questionnaire to ensure they met the inclusion and exclusion criteria. Each participant was evaluated by two different examiners, with the examiner order randomly assigned on the first day and reversed on the second day. Neither examiner had access to the other’s results or their own previous data. A third examiner was responsible for securely storing the results. Additionally, participants were not informed of their prior evaluation outcomes. Tests were separated by rest intervals of 5–15 min, with approximately 14 days between the first and second sessions [35,40].

### 2.5. Statistical Analysis

Data analysis was conducted using SPSS^®^ version 21.0 for Windows (Statistical Package for the Social Sciences, Inc., Chicago, IL, USA). To evaluate the influence of sex, an analysis of covariance (ANCOVA) was performed, while Pearson correlation was used to assess the influence of age. In both cases, general average values were used to obtain a more stable estimate of the true measure of the participant [17]. A General Linear Model (GLM) for repeated measures was applied, with general and specific saccadic time values as the dependent variable and examiner and session as independent variables, to detect differences in mean saccadic time.

Intraexaminer repeatability and interexaminer reproducibility were assessed through within-participant standard deviation and 95% confidence limits using one-way ANOVA [28]. Intraclass correlation coefficients (ICCs) and their 95% confidence intervals (CIs) were calculated to determine intra- and inter-rater reliability using a two-way mixed absolute agreement model [41]. ICC values were interpreted as follows: values ≤ 0.40 indicates poor repeatability; values > 0.40 and < 0.75 indicate moderate to good repeatability; values ≥ 0.75 indicate good to excellent repeatability [17]. Bland–Altman analysis [42] was employed to establish the limits of agreement, summarizing the bias, standard deviation of differences, 95% limits of agreement (LoA), and confidence limits around the LoA.

## 3. Results

### 3.1. Participants

**Figure 1:**
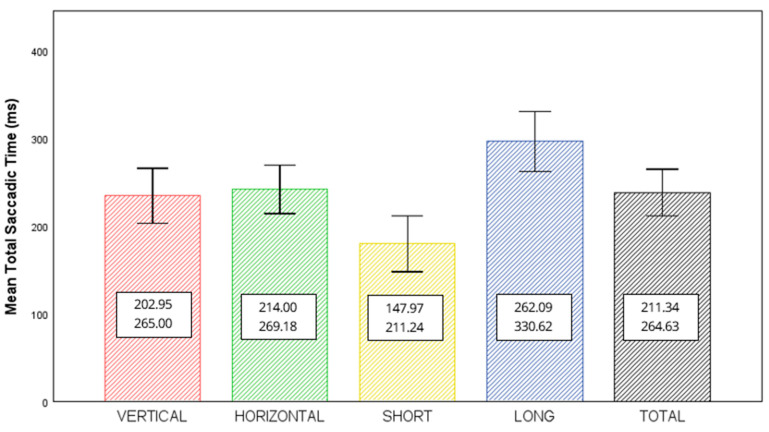
Mean values (±standard deviation) of mean saccadic time (total, vertical, horizontal, short and long) measured using COI-SV^®^ software. Numeric values indicate the minimum and maximum observed for each condition.

A total of 78 participants were included in this study (41 men and 37 women), with a mean age of 29.5 years (range: 19–64). Twenty-five participants (32%) did not attend the second visit, leaving 53 participants for the intersession analysis. Baseline comparisons of age and sex between completers and non-completers showed no significant differences. Regarding refractive status, 48 participants were emmetropic (61.5%), 19 were myopic (24.4%), 10 were hyperopic (12.8%), and 1 was astigmatic (1.3%). Mean saccadic times were 179.61 ± 31.64 ms for short, 296.35 ± 34.26 ms for long, 234.38 ± 31.43 ms for vertical, 241.59 ± 27.59 ms for horizontal, and 237.98 ± 26.65 ms for total mean values. Figure 1 displays the average saccadic time for each of the general items calculated.

### 3.2. Influence of Age and Sex

Since the groups were not age-matched, ANCOVA (analysis of covariance) was employed to determine the relationship between the mean saccadic values and sex, concluding that there are no significant differences between men and women in all general measures (*p*-values > 0.05). Regarding age, it was used Pearson correlation to determine if saccadic time values could be related to age, concluding significantly (*p*-values ≤ 0.05), that both parameters had a direct relationship, so that the older the participant was, the worse saccadic duration values were (longer times).

### 3.3. Estimates of Repeatability and Reproducibility

Repeatability and reproducibility were assessed using three methods: a general linear model with repeated measures, ANOVA with its variability and confidence intervals and ICCs with its confidence intervals. Each of these methods was applied to both specific and general saccadic time values and can be seen in Table 1 and Table 2.

**Figure 2:**
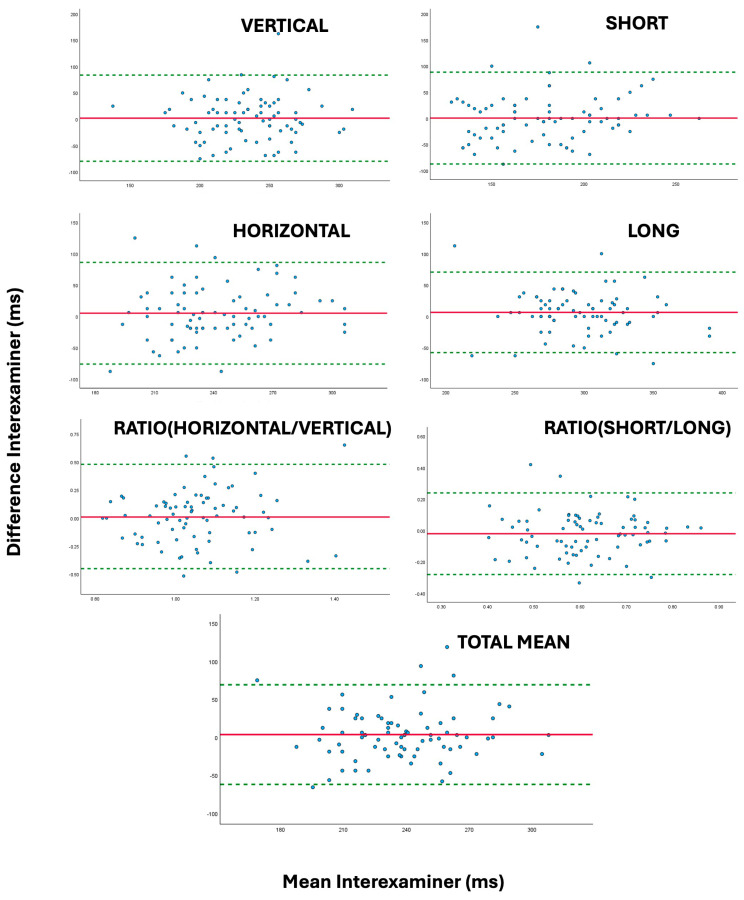
Bland–Altman plots illustrating the level of agreement in saccadic time values (Vertical, Horizontal, Ratio Vertical–Horizontal, Short, Long, Ratio Short–Long, Total Mean) between examiner 1 and examiner 2. The solid red lines indicate the interexaminer mean difference, while the dotted green lines represent the 95% confidence intervals for the limits of agreement.

**Figure 3:**
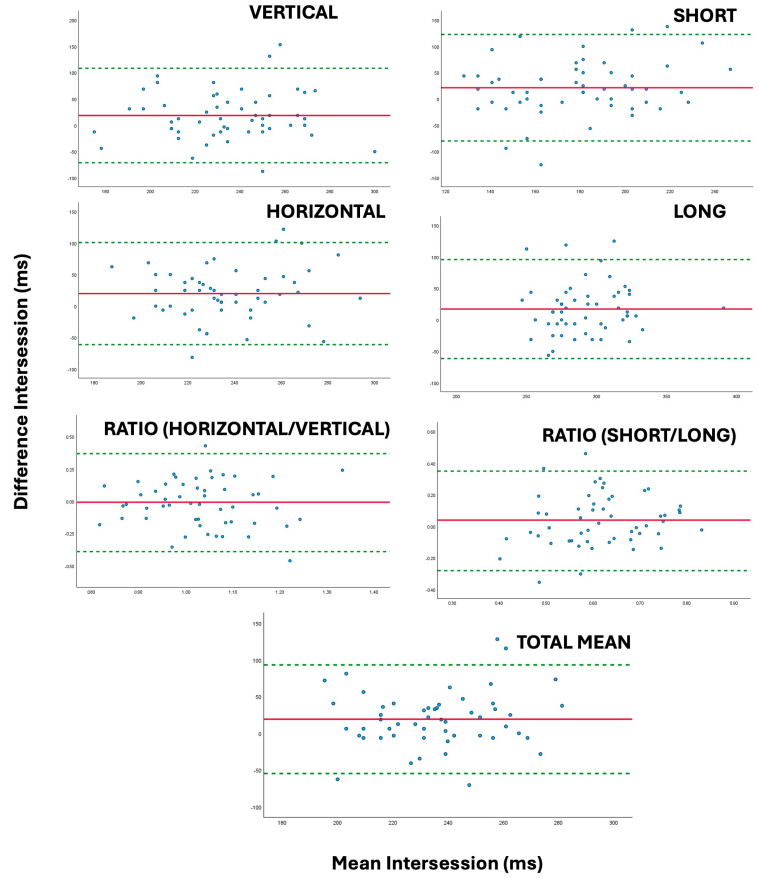
Bland–Altman plots illustrating the level of agreement in saccadic time values (Vertical, Horizontal, Ratio Vertical–Horizontal, Short, Long, Ratio Short–Long, Total Mean) between session 1 and session 2. The solid red lines indicate the intersession mean difference, while the dotted green lines represent the 95% confidence intervals for the limits of agreement.

The Bland–Altman analysis showed overall bias was small (<50 ms), with limits of agreement ranging from −159.0 and 220.3 ms for specific saccadic speed values and from −87.0 to 122.52 ms in case of general values, which could be useful in clinical practice. Figure 2 and Figure 3 show Bland–Altman plots for interexaminer and intersession comparisons of general saccadic time values.

The graphs in Figure 2 and Figure 3 illustrate the level of agreement between the different saccadic time measurements; narrower intervals reflect better repeatability across sessions or examiners. In both figures, the figures appear symmetrically distributed around the mean difference, suggesting that variability in repeated saccadic time measurements across sessions is mainly random rather than systematic.

## 4. Discussion

Our study provided estimates of the repeatability of a method designed to measure saccadic time in healthy participants, determining the precision of the software (COI-SV^®^) to distinguish a true clinical change from measurement error [43].

Accurate measurement of saccadic time in each direction is important for sports vision and other field. Objective methods offer important benefits, but their repeatability must be established. To date, no data exists on the use of the COI-SV^®^ to obtain saccadic time values.

Regarding average values, they may be clinically useful. As there are no prior data on the use of the software to measure saccadic time, the reference values obtained in this study could serve as preliminary normative benchmarks to guide future research. Research by Stan et al. [16] used a similar task involving a red dot as stimulus, with a target duration ranging from 1000 to 1200 ms, allowing participants to complete the task within the required time. In contrast, our protocol also required letter recognition, thus integrating perceptual and cognitive processing in addition to motor execution. This dual demand may account for the longer mean saccadic durations we observed, extending previous work by showing how recognition components increase task complexity. Another study that evaluated saccade duration reported values between 31 and 68 ms using an eye tracker [23], but this purely motor measure is not directly comparable to our findings. Similarly, a cognitive-task protocol focusing on older adults (50–80 years) found reaction times between 349 and 363 ms [44]. Our results fall between these ranges, suggesting that the combination of perceptual recognition and younger, mixed-age participants yields intermediate values, thereby providing new insights into the influence of task design and population characteristics on saccadic performance. Additionally, studies assessing only latency for neurological disorders [25] cannot be directly compared, as our focus was on functional measures relevant to sports vision.

Saccadic time has also been investigated in driving tasks, where researchers observed different values between drivers with no accident history and those previously involved in accidents [26]. Differences in saccade time have also been found in patients with a history of concussion, evaluated with an eye tracker [24]. Other studies have incorporated eye tracker while performing DEM and King-Devick test, obtaining data on saccadic speed and duration [22,45,46]. However, we cannot compare them with our research, as these tests include simulated reading tasks that require low-amplitude saccades. The key findings from these studies include an inverse relationship between saccade performance and intellectual disability [22], as well as an inverse relationship between saccade performance and task duration [46].

The main importance of this study lies in the moderate repeatability of saccadic duration measurements, using COI-SV^®^ software, suggesting that the test can be reliably administered by any examiner. Regarding specific measures, 6 out of 8 ICC values demonstrated moderate to good reliability, although the lower limit of agreements indicated poor reliability (<0.400) in 7 out of 8 ICC values. Importantly, some ICC confidence intervals included negative lower bounds, which indicate extremely poor reliability and further highlight the limitations of the method. Additionally, the Bland–Altman analysis showed limits of agreement ranging from −159.0 to 220.3 ms for specific measures and from −87.0 to 122.52 ms for general measures (Table 1 and Table 2). These correspond to interval widths of 201.2 to 379.3 ms for specific values and 128.2 to 202.35 ms for general values. However, the wide dispersion of these limits (up to ±220 ms) also suggests substantial variability, which restricts their direct applicability in clinical decision-making and should be interpreted with caution.

Regarding intra-observer repeatability, the study aimed to determine whether the data were objective enough to minimize significant variation caused by factors unrelated to the test, such as the participant’s mental state, time of day, or fatigue, as suggested by previous research [17,46]. ICC values of specific measures demonstrated only 2 out of 8 ICC values indicated moderate to good reliability with lower limit of agreements being less than 0.400 in all measures. Regarding general measures, only 1 out of 7 measures demonstrated moderate to good reliability. For that reason, we cannot conclude that intersession repeatability was high. Based on these findings, an ANOVA was conducted to explore the sources of variability. The analysis revealed that 8 out of 15 measures (3 specific and 5 general) showed statistically significant differences between sessions, with performance consistently better in the second session. Unrelated factors may have contributed to these differences; further research is needed.

One possible hypothesis is the presence of a learning effect. To investigate this, ANOVA was performed to compare the results from the first and second trials within the same session, revealing no significant differences in 7 out of 8 of the specific saccadic measurements. Consequently, it remains unclear whether a slight learning effect occurred and could be considered a limitation of the study. This potential learning effect could have contributed to better performance in the second trial by reducing variability and artificially improving repeatability. To minimize this bias, future studies should consider incorporating extended familiarization trials, counterbalancing trial order, or adding longer intervals between trials to better control for learning effects. We attempted to control for this by randomizing the order of examiners, following the methodology of other similar studies [35,36]. To improve this issue, future studies could incorporate a more accurate and extended pre-test, ensuring that participants do not become familiar with the test once the measurements are taken. In comparison with other similar studies, some acknowledged a mild learning effect [37,47], while others did not address this issue [39].

As for the influence of age and sex, our findings are consistent with those of Bowling et al. [44], who reported that older individuals exhibited slower saccade reaction times with a moderate correlation. We similarly observed a direct association between age and longer saccadic durations, with correlation coefficients in the low-to-moderate range (r ≈ 0.25–0.35), indicating modest but significant effects. Our study advances this evidence by applying a digital, time-sensitive tool in a sports vision context, showing that age-related slowing is detectable even in a healthy, non-clinical population. Likewise, Song et al. [27] demonstrated that older participants had significantly fewer fixations and poorer saccade reaction times during the DEM test. They also found sex-related differences in fixation counts, though not in saccadic speed, which aligns with our results. Together, these comparisons confirm the robustness of the age effect while highlighting that sex-related differences in saccadic duration remain limited. Given the scarcity of studies in athletic or non-clinical cohorts, our work provides novel evidence and underscores the need for further research in broader populations.

In terms of methodology, we used a similar protocol to that of Rider et al. [35]. A key strength of the study was the use of a rigorous and standardized protocol, which included uniform participant instructions, consistent room conditions, and a randomized examiner sequence to minimize variability. In addition, the inclusion of both intraobserver and interobserver repeatability, along with consideration of the inter-visit factor, enhances the strength of our study when compared to similar repeatability studies, which often focus solely on interobserver repeatability [39,41,42] or intraobserver repeatability [47,48]. Furthermore, our sample size was larger than that of other comparable studies [37,38,47,48,49].

From a broader perspective, these findings may have practical relevance in the field of sports vision, where rapid and accurate saccadic movements are linked to visual performance in tasks such as ball tracking, anticipation, and decision-making. Establishing normative values with COI-SV^®^ could aid in designing training or rehabilitation protocols tailored to athletes, as well as in monitoring their progress over time. This study has several limitations. First, 25 participants (32% of the original sample) did not attend the second visit, which reduced the sample size for the intersession analysis and may have affected the statistical power of our findings. Baseline comparisons showed no significant differences in age or sex between completers and non-completers; however, it is possible that this dropout introduced attrition bias if certain groups (e.g., older participants or those with slower performance) were less likely to return. Future studies should consider strategies to improve participant retention, such as shorter intervals between visits, flexible scheduling, or regular reminders and follow-up contacts.

Second, although the test was originally designed to assess athletes, the sample included very few participants over the age of 50. This limits the generalizability of our findings to older populations, where age-related slowing of saccades is likely more pronounced. Recruiting a larger and more age-diverse cohort would strengthen future research and allow normative values to be established across age groups.

Finally, although a familiarization trial was included to reduce learning effects, some variability between sessions may still have been influenced by this factor. Longer or repeated familiarization phases may help to minimize potential learning effects in future work.

This study holds particular significance as, at present, optometrists do not have standardized methods to compare optometric variables in high-performance athletes. The COI-SV^®^ software could be a highly valuable tool for optometrists working in the field of sports vision. Beyond its potential as a measurement instrument, the establishment of normative values and repeatability indices can inform the development of individualized training programs aimed at enhancing ocular motility and perceptual–motor integration. In clinical practice, these findings may support the use of saccadic duration assessments to identify athletes with slower visual processing, enabling targeted vision training protocols and monitoring progress over time. Furthermore, the incorporation of reliable saccadic testing into pre-season screenings or return-to-play evaluations could provide a more objective framework for assessing visual performance in athletes and guiding tailored rehabilitation strategies.

## 5. Conclusions

In conclusion, this study provides the first evidence on the repeatability of saccadic duration measurements obtained with the COI-SV^®^ software. Our results showed moderate to good interexaminer reliability but poor intraexaminer repeatability, which underscores both the potential and the current limitations of the method. These findings provide additional evidence supporting the test’s validity, intraexaminer reliability remains insufficient, suggesting that further improvements and validation are required before any clinical adoption. Importantly, the normative values reported here may serve as a benchmark for future studies, and the feasibility of using a time-sensitive, digital tool in sports vision highlights its potential clinical relevance. Future research should focus on optimizing the protocol, improving examiner consistency, and extending validation to larger, age-diverse, and athletic populations, so that this tool can ultimately contribute to more comprehensive visual examinations in both clinical and sports settings.

## Figures and Tables

**Table 1:** Summary of the most important statistics in terms of inter and intraexaminer reliability applied to specific time values (Up 20°, Up 10°, Left 30°, Left 15°, Right 15°, Right 30°, Down 10°, Down 20°).

TEST	Interexaminer Repeatability (Δ) (95% Confidence Limits)	Intersession Repeatability (Δ) (95% Confidence Limits)	ANOVA Interexaminer (*p*-Valor)	ANOVA Intersession (*p*-Valor)	Interexaminer Repeatability (ICC) (95% Confidence Limits)	Intersession Repeatability (ICC) (95% Confidence Limits)
UP 20°	1.28 (−112.6 to 115.2)	7.55 (−93.1 to 108.1)	0.846	0.289	0.591 (0.357 to 0.740)	0.524 (0.179 to 0.725)
UP 10°	2.88 (−136.2 to 141.9)	19.34 (−92.2 to 130.8)	0.720	0.017	0.203 (−0.256 to 0.494)	0.476 (0.116 to 0.693)
LEFT 30°	4.17 (−105.7 to 114.1)	12.26 (−91.4 to 115.9)	0.514	0.097	0.445 (0.128 to 0.646)	0.377 (−0.060 to 0.636)
LEFT 15°	−7.05 (−149.4 to 135.3)	20.75 (−141.1 to 182.6)	0.394	0.073	0.500 (0.216 to 0.681)	0.101 (−0.512 to 0.472)
RIGHT 15°	10.58 (−137.3 to 158.4)	14.62 (−127.4 to 156.6)	0.219	0.148	0.240 (−0.187 to 0.514)	0.144 (−0.460 to 0.502)
RIGHT 30°	13.14 (−108.2 to 134.5)	32.08 (−90.5 to 154.6)	0.065	<0.001	0.539 (0.283 to 0.704)	0.184 (−0.269 to 0.496)
DOWN 10°	−3.53 (−145.0 to 137.9)	30.66 (−159.0 to 220.3)	0.667	0.025	0.592 (0.358 to 0.740)	0.131 (−0.432 to 0.484)
DOWN 20°	7.69 (−105.1 to 120.5)	16.98 (−124.3 to 158.2)	0.241	0.092	0.659 (0.467 to 0.782)	0.229 (−0.306 to 0.549)

Note: *p*-values lower than 0.05 (statistically significant differences) are shown in red. ICCs values higher than 0.4 (moderate to good repeatability) are shown in orange.

**Table 2:** Summary of the most important statistics in terms of inter and intraexaminer reliability applied to general time values (Vertical, Horizontal, Horizontal/Vertical Ratio, Short, Long, Short/Long Ratio, Total Mean).

TEST	Interexaminer Repeatability (Δ) (95% Confidence Limits)	Intersession Repeatability (Δ) (95% Confidence Limits)	ANOVA Interexaminer (*p*-Valor)	ANOVA Intersession (*p*-valor)	Interexaminer Repeatability (ICC) (95% Confidence Limits)	Intersession Repeatability (ICC) (95% Confidence Limits)
VERT	2.08 (−79.8 to 83.9)	18.63 (−71.1 to 108.4)	0.661	0.005	0.561 (0.310 to 0.721)	0.205 (−0.281 to 0.520)
HORIZ	5.21 (−75.8 to 86.2)	19.93 (−61.5 to 100.9)	0.269	<0.001	0.439 (0.121 to 0.641)	0.183 (−0.281 to 0.498)
RATIOhv	0.02 (−0.45 to 0.48)	−0.01 (−0.39 to 0.37)	0.548	0.803	0.065 (−0.472 to 0.406)	0.201 (−0.398 to 0.542)
SHORT	0.72 (−87.0 to 88.5)	21.34 (−79.83 to 122.52)	0.887	0.004	0.502 (0.217 to 0.683)	0.196 (−0.290 to 0.514)
LONG	6.57 (−57.5 to 70.7)	17.22 (−61.3 to 95.7)	0.080	0.003	0.767 (0.636 to 0.852)	0.410 (0.019 to 0.651)
RATIOSL	−0.02 (−0.28 to 0.35)	0.04 (−0.28 to 0.35)	0.25	0.106	0.598 (0.371 to 0.743)	0.343 (−0.118 to 0.617)
TOTAL MEAN	3.65 (−61.8 to 69.1)	19.28 (−54.6 to 93.1)	0.34	<0.001	0.607 (0.384 to 0.749)	0.211 (−0.235 to 0.515)

Note: *p*-values lower than 0.05 (statistically significant differences) are shown in red. ICCs values higher than 0.4 (moderate to good repeatability) are shown in orange and higher than 0.75 (good to excellent repeatability) are shown in green.

## Data Availability

The raw data supporting the conclusions of this article will be made available by the authors on request.
